# Medical and Health-Related Misinformation on Social Media: Bibliometric Study of the Scientific Literature

**DOI:** 10.2196/28152

**Published:** 2022-01-25

**Authors:** Andy Wai Kan Yeung, Anela Tosevska, Elisabeth Klager, Fabian Eibensteiner, Christos Tsagkaris, Emil D Parvanov, Faisal A Nawaz, Sabine Völkl-Kernstock, Eva Schaden, Maria Kletecka-Pulker, Harald Willschke, Atanas G Atanasov

**Affiliations:** 1 Oral and Maxillofacial Radiology, Applied Oral Sciences and Community Dental Care Faculty of Dentistry The University of Hong Kong Hong Kong China; 2 Ludwig Boltzmann Institute for Digital Health and Patient Safety Medical University of Vienna Vienna Austria; 3 Department of Molecular Cell and Developmental Biology University of California Los Angeles Los Angeles, CA United States; 4 Division of Pediatric Nephrology and Gastroenterology Comprehensive Center for Pediatrics Medical University of Vienna Vienna Austria; 5 Faculty of Medicine University of Crete Heraklion Greece; 6 Department of Translational Stem Cell Biology Medical University of Varna Varna Bulgaria; 7 College of Medicine Mohammed Bin Rashid University of Medicine and Health Sciences Dubai United Arab Emirates; 8 Department of Anaesthesia, Intensive Care Medicine and Pain Medicine Medical University of Vienna Vienna Austria; 9 Institute of Genetics and Animal Biotechnology of the Polish Academy of Sciences Jastrzebiec Poland

**Keywords:** COVID-19, Twitter, health, social media, bibliometric, dissemination, knowledge exchange

## Abstract

**Background:**

Social media has been extensively used for the communication of health-related information and consecutively for the potential spread of medical misinformation. Conventional systematic reviews have been published on this topic to identify original articles and to summarize their methodological approaches and themes. A bibliometric study could complement their findings, for instance, by evaluating the geographical distribution of the publications and determining if they were well cited and disseminated in high-impact journals.

**Objective:**

The aim of this study was to perform a bibliometric analysis of the current literature to discover the prevalent trends and topics related to medical misinformation on social media.

**Methods:**

The Web of Science Core Collection electronic database was accessed to identify relevant papers with the following search string: ALL=(misinformati* OR “wrong informati*” OR disinformati* OR “misleading informati*” OR “fake news*”) AND ALL=(medic* OR illness* OR disease* OR health* OR pharma* OR drug* OR therap*) AND ALL=(“social media*” OR Facebook* OR Twitter* OR Instagram* OR YouTube* OR Weibo* OR Whatsapp* OR Reddit* OR TikTok* OR WeChat*). Full records were exported to a bibliometric software, VOSviewer, to link bibliographic information with citation data. Term and keyword maps were created to illustrate recurring terms and keywords.

**Results:**

Based on an analysis of 529 papers on medical and health-related misinformation on social media, we found that the most popularly investigated social media platforms were Twitter (n=90), YouTube (n=67), and Facebook (n=57). Articles targeting these 3 platforms had higher citations per paper (>13.7) than articles covering other social media platforms (Instagram, Weibo, WhatsApp, Reddit, and WeChat; citations per paper <8.7). Moreover, social media platform–specific papers accounted for 44.1% (233/529) of all identified publications. Investigations on these platforms had different foci. Twitter-based research explored cyberchondria and hypochondriasis, YouTube-based research explored tobacco smoking, and Facebook-based research studied vaccine hesitancy related to autism. COVID-19 was a common topic investigated across all platforms. Overall, the United States contributed to half of all identified papers, and 80% of the top 10 most productive institutions were based in this country. The identified papers were mostly published in journals of the categories public environmental and occupational health, communication, health care sciences services, medical informatics, and medicine general internal, with the top journal being the Journal of Medical Internet Research.

**Conclusions:**

There is a significant platform-specific topic preference for social media investigations on medical misinformation. With a large population of internet users from China, it may be reasonably expected that Weibo, WeChat, and TikTok (and its Chinese version Douyin) would be more investigated in future studies. Currently, these platforms present research gaps that leave their usage and information dissemination warranting further evaluation. Future studies should also include social platforms targeting non-English users to provide a wider global perspective.

## Introduction

Public health information has been traditionally distributed to the public with the use of printed media, television, or radio. With the rise of participatory web and social media [1] and particularly in the face of recent pandemics, such as the H1N1 influenza pandemic in 2009 and the COVID-19 pandemic [2], the internet plays a major role in information sharing. The general public no longer acts as a passive consumer but plays a critical role in the generation, filtering, and amplification of public health information [1]. Health care–related scientific discoveries are now often condensed into news pieces written in layman’s terms and disseminated to broad and nonexpert audiences via social media, which contributes to not only better visibility of important information, but also better communication between health care professionals and the community [3]. Another major benefit of social media for health care is the potential for patient empowerment by providing a platform where patients can get information about their medical condition, communicate with health care professionals, share their experiences, and support other individuals affected by the same condition [4].

While providing numerous empowerment opportunities, there lies a great potential for miscommunication and misinformation [5] within the social media–based setting of health-related information distribution. While social media has increased and improved the dissemination of scientific results to the community, it has also increased the sensationalist language used to describe scientific findings [6,7]. Often, media articles may report research findings with misinterpretation and overstatement that can lead to confusion, misinformation, and mistrust in scientific reporting [6]. Moreover, social media empowers pseudoexperts and nonexpert influencers in sharing opinions and false information in the area of health care [8]. Very often, important societal figures, such as celebrities, politicians, and activists, without any expert knowledge of a certain topic, but with a large influence, can take part in spreading health-related misinformation [8]. The need for social media to moderate the information shared and increase expert consultation is increasingly evident and could be one way to reduce the spread of misinformation [9].

One of the most polarizing topics in recent years has been vaccination, following a scientific article from 1998 by Wakefield et al, which proposed a causative link between the measles, mumps, and rubella (MMR) vaccine and autism [3,10]. The study by Wakefield et al was later found to be flawed and fraudulent and was retracted [11-14]. Even though the findings in the study by Wakefield et al have since been disproved as numerous subsequent studies found no link between vaccines and autism, the study caused great damage to vaccine programs worldwide, with a considerable increase in the number of people rejecting vaccination in the past decades [9]. Another prominent illustrative example is the case of measles reappearance in the United States [15]. In the United States, there was an immense surge in antivaccine Tweets around 2015 to 2016, closely following the 2014 to 2015 measles outbreak and the release of Wakefield’s antivaccine movie *Vaxxed* in 2016 [16]. This could be linked to the finding that antivaccine posts on Facebook were often shared and liked more often than provaccine posts [17]. Similarly, individuals exposed to negative opinions on vaccination are more likely to further share the opinions compared to individuals exposed to positive or neutral opinions [18]. This is potentiated by the so called “echo chamber effect,” where many social media users are exposed to curated content that is likely to align to their existing beliefs and exacerbates the strength of the misinformation they receive [19,20].

As medical misinformation is an increasingly relevant topic to study, the amount of available literature is growing. On this background, the aim of this study was to perform a bibliometric analysis of the current literature to discover the prevalent trends and topics related to medical misinformation on social media. Conventional systematic reviews have been published on this topic to identify original articles and summarize their methodological approaches and themes [7,21]. A bibliometric study could complement their findings, for instance, by identifying the most productive authors and institutions, evaluating the geographical distribution of the publications, revealing recurring journals disseminating such research findings, unveiling the most common keywords or concepts reported, and evaluating if the publications were well cited and disseminated in journals with high impact factors. These data can serve as starting points to guide fellow researchers to pinpoint papers relevant to their studies, contact potential collaborators to conduct joint research, and find suitable journals to submit their work. At the same time, these data can help researchers find missing gaps in the literature and missing parties contributing to the field, so that the missing pieces can be filled. Since the most common social media platforms have originated in the United States, it was hypothesized that the United States would have the highest contribution in this area of academic research. This would be an important research question as publication bias toward the United States might shadow or fail to capture the wider spectrum of global developments and experiences regarding medical misinformation on social media.

## Methods

### Data Source and Search Strategy

A bibliometric analysis is a study that applies mathematical and statistical methods to books and other media of communication, such as academic publications [22]. Similar to a previous bibliometric study [23], this work was performed in accordance with the Preferred Reporting Items for Systematic Reviews and Meta-Analysis (PRISMA) statement [24]. The Web of Science (WoS) Core Collection database was accessed on January 13, 2021, via the following search string: ALL=(misinformati* OR “wrong informati*” OR disinformati* OR “misleading informati*” OR “fake news*”) AND ALL=(medic* OR illness* OR disease* OR health* OR pharma* OR drug* OR therap*) AND ALL=(“social media*” OR Facebook* OR Twitter* OR Instagram* OR YouTube* OR Weibo* OR Whatsapp* OR Reddit* OR TikTok* OR WeChat*). The PubMed database was similarly searched for papers mentioning these terms in their titles and abstracts. The search terms about misinformation and its common synonyms were referred from 2 recent systematic reviews [7,25]. No additional filter was placed to restrict the search results, and the indicated search yielded 529 papers in WoS and 285 papers in PubMed. After merging the lists from both databases and removing duplicates, 529 papers remained. Since this was a total-scale analysis of the concerned literature [26], all resultant papers were included without exclusion (Multimedia Appendix 1).

The “Analyze” function of WoS was used to provide initial descriptive statistics regarding the bibliographic data. The numbers of social media platform–specific papers were counted. The approach applied to Facebook is presented here as an example for the used evaluation strategy. In particular, we additionally searched with the following search string: ALL= Facebook* NOT (Twitter* OR Instagram* OR YouTube* OR Weibo* OR Whatsapp* OR Reddit* OR TikTok* OR WeChat*). When the original search string and this new search string were combined with the Boolean operator “AND,” the resulting papers mentioned Facebook but not the other referenced social media.

### Outcome Measures

We evaluated the publication and citation counts of contributors in terms of author, institution, country, and journal. We also computed the publication and citation counts of terms and keywords, and identified the top 10 most cited papers. The semantic content of the identified publications was analyzed in the following ways. Citations per paper (CPPs) were computed for terms occurring in the titles, abstracts, and keywords of the identified papers, and n-gram analysis was conducted to identify the most recurring metatext. These analyses aimed to answer the queries listed at the end of the Introduction. Further details are described below.

### Data Extraction and Main Analysis

The 529 identified papers were exported in full record with cited references to VOSviewer [27,28] for subsequent bibliometric analyses and visualizations. To visualize the results, a term map was created via VOSviewer to display publication and citation data for terms that appeared in the titles and abstracts of the analyzed papers. We decided to visualize terms that appeared in over 1% of the papers (ie, at least six papers) for improved clarity of the generated image, to avoid a heavily crowded figure [26]. A keyword map was similarly produced with the same frequency threshold, displaying author keywords and keywords added by WoS (KeyWords Plus) altogether. VOSviewer performs text mining by part-of-speech tagging with the aid of Apache OpenNLP and a linguistic filter, and converts plural noun phrases into singular form [29]. Meanwhile, it constructs a map in the following 3 steps based on a co-occurrence matrix: (1) calculation of a similarity index based on association strength (also known as proximity index and probabilistic affinity index), (2) application of the VOS mapping technique to the matrix, and (3) solution transformation to produce consistent results [27]. Besides visualizations, the resultant data from the maps were checked, and the recurring items were presented in tabular format.

In addition, keyword maps were produced for subsets of papers that were specific to Twitter, YouTube, and Facebook. For these maps, keywords with at least two appearances were included.

### Exploratory Analysis

Finally, an exploratory n-gram analysis was conducted with the online NGram Analyzer [30] that allows n-gram metatexts to be listed. The abstracts of the publications were pasted into the program and the recurring 5-grams (a contiguous sequence of 5 words) were extracted. After manual checking, meaningful 5-grams with at least four appearances have been reported in the Results.

## Results

**Figure 1:**
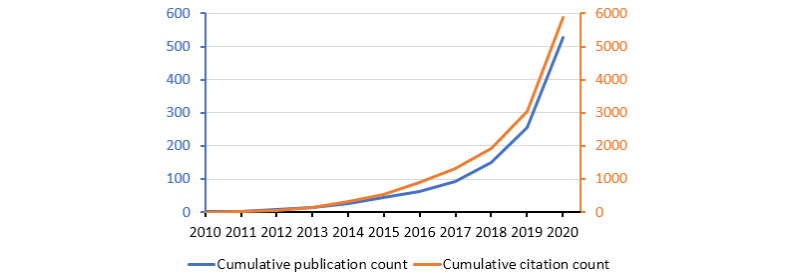
Total publication and citation counts of papers on medical and health-related misinformation on social media. Data are shown until the end of 2020.

Our search strategy identified a total of 529 scientific articles addressing medical misinformation on social media. The analysis of these papers revealed that the earliest papers on this subject could be traced back to 2010 and 2011, and the total publication and citation counts increased very rapidly, especially during the last 2 years (Figure 1). Original articles accounted for the majority of the identified publications (n=393, 74.3%), followed by editorial materials (n=50, 9.5%). The article-to-review ratio was 12.7:1 (n=393 vs 31). Proceedings accounted for another 7.2% (n=38). Over 97% of the indexed papers were written in English. The most cited paper among the 529 was also the oldest paper; it involved content analysis of over 5000 relevant Tweets during the 2009 H1N1 outbreak [1]. Within a decade, it has already accumulated 589 citations.

The most productive author publishing in this subject area was Emily K Vraga from George Mason University (Virginia, USA). She started to publish on this topic in 2015 and accumulated a total of 13 papers, mostly with Leticia Bode and Melissa Tully. Leticia Bode and Joseph A Hill followed second in the list of the most productive researchers, with 9 papers each. Following them were 27 authors with 7 papers each. The top 10 most productive institutions, countries, journals, and WoS categories in which the analyzed works were published are listed in Table 1. The United States contributed to half (265/529, 50.1%) of the identified papers and was the home country of 80% of the top 10 most productive institutions. The identified papers were mostly published in journals belonging to the following categories: public environmental and occupational health, communication, health care sciences services, medical informatics, and medicine general internal.

Social media platform–specific papers accounted for 44.1% (n=233) of all 529 identified papers (Table 2). The most popularly investigated social media were Twitter, YouTube, and Facebook. They also had higher CPPs than other social media.

**Table 1:** Top 10 most productive institutions, countries, journals, and Web of Science categories publishing papers on medical and health-related misinformation on social media.

Variable		Publication count (N=529), n (%)	Citations per paper
**Institution**			
	Harvard University	25 (4.7)	13.2
	University of Texas System	20 (3.8)	3.4
	University of North Carolina	14 (2.6)	13.8
	University of Pennsylvania	14 (2.6)	11.9
	University of London	13 (2.5)	30.5
	Johns Hopkins University	12 (2.3)	6.0
	University of California System	11 (2.1)	1.7
	University of Minnesota System	11 (2.1)	2.8
	Pennsylvania Commonwealth System of Higher Education	10 (1.9)	3.6
	University System of Sydney	10 (1.9)	32.1
**Country**			
	United States	265 (50.1)	12.2
	United Kingdom	53 (9.3)	20.0
	Italy	35 (6.6)	9.2
	Canada	33 (6.2)	34.0
	Spain	30 (5.7)	7.2
	Australia	27 (5.1)	19.0
	China	27 (5.1)	13.7
	Turkey	17 (3.2)	5.1
	Germany	15 (2.8)	27.9
	India	14 (2.6)	2.8
	Switzerland	14 (2.6)	14.9
**Journal (2019 impact factor)**			
	Journal of Medical Internet Research (5.034)	32 (6.0)	14.1
	American Journal of Public Health (6.464)	14 (2.6)	3.1
	Health Communication (1.965)	13 (2.5)	9.2
	Vaccine (3.143)	13 (2.5)	28.1
	International Journal of Environmental Research and Public Health (2.468)	11 (2.1)	8.6
	PLOS One (2.740)	11 (2.1)	60.1
	Annals of Behavioral Medicine (4.475)	8^a^ (1.5)	0
	Professional de la Informacion (N/A^b^)	8 (1.5)	8.6
	Cureus (N/A)	6 (1.1)	26.7
	Journal of Health Communication (1.596)	6 (1.1)	2.3
**Web of Science category**			
	Public environmental and occupational health	95 (18.0)	12.6
	Communication	71 (13.4)	7.3
	Health care sciences services	50 (9.5)	17.5
	Medical informatics	48 (9.1)	17.4
	Medicine general internal	38 (7.2)	17.2
	Computer science information systems	33 (6.2)	4.1
	Information science library science	32 (6.0)	5.5
	Health policy services	22 (4.2)	13.6
	Computer science theory methods	21 (4.0)	5.4
	Immunology	21 (4.0)	23.6

^a^All 8 publications in Annals of Behavioral Medicine were meeting abstracts and received no citation.

^b^N/A: not applicable.

**Table 2:** Count of platform-specific papers on medical and health-related misinformation on social media.

Social media	Publication count, n	Citations per paper
Twitter	90	17.0
YouTube	67	13.7
Facebook	57	15.3
WhatsApp	6	4.0
Instagram	6	8.7
Weibo	4	7.5
Reddit	2	2.5
WeChat	1	3.0
TikTok	0	N/A^a^

^a^N/A: not applicable.

**Figure 2:**
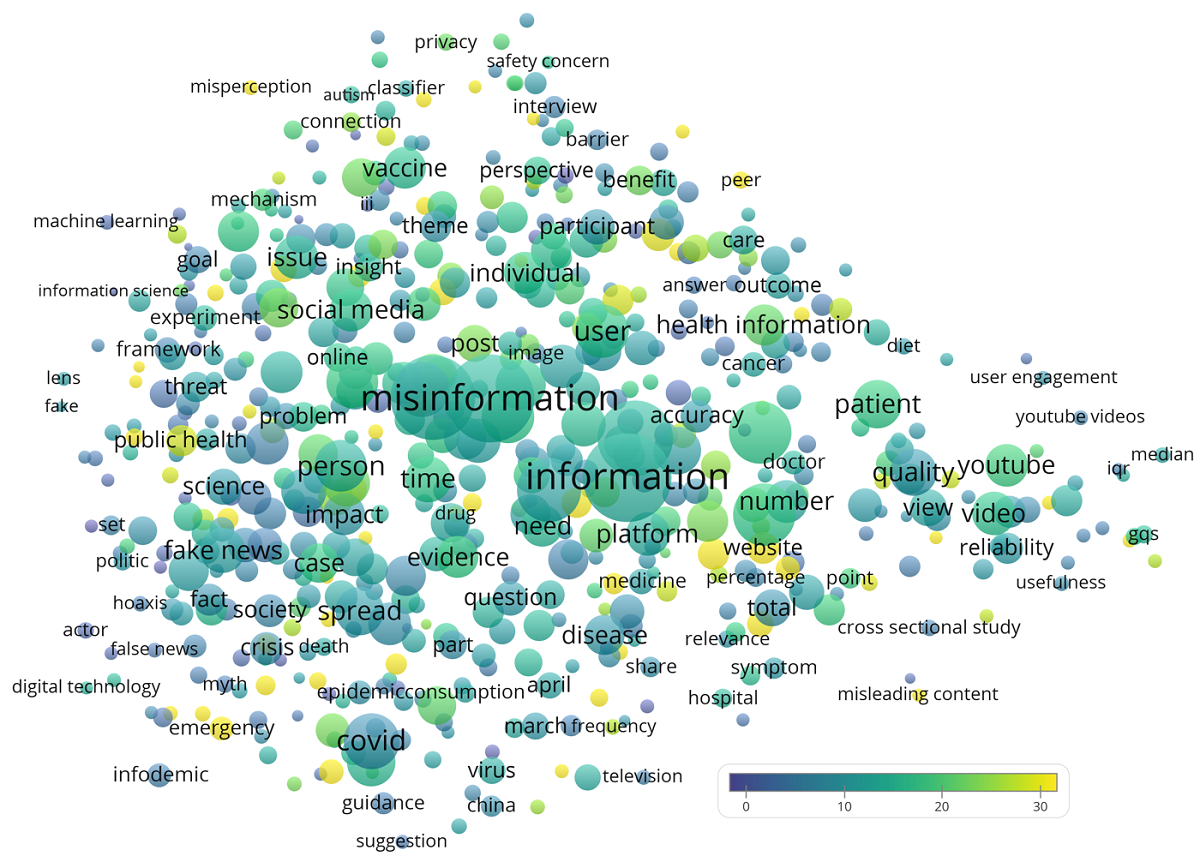
Term map showing words/phrases extracted from the titles and abstracts of the 529 papers. Circle size is related to the number of papers mentioning the word/phrase. Circle color is related to the citations per paper. The proximity between circles is related to how frequently the terms are co-mentioned in the same papers.

Figure 2 shows the terms extracted from the titles and abstracts of all 529 identified papers. COVID-19 (“covid” at the lower half, n=109, CPP=7.1) and vaccine (upper half, n=62, CPP=15.7) were 2 major health issues identified. Mentioned COVID-19 derivatives included SARS-CoV-2 (“sars cov,” n=9, CPP=11.0), coronavirus (n=22, CPP=15.3), coronavirus disease (n=25, CPP=12.6), and coronavirus pandemic (n=6, CPP=2.3). Mentioned vaccine derivatives included vaccination (n=53, CPP=21.6), vaccination rate (n=7, CPP=5.7), vaccine hesitancy (n=14, CPP=13.8), vaccine misinformation (n=13, CPP=6.9), vaccine preventable disease (n=6, CPP=29.0), and vaccine safety (n=8, CPP=7.8). The top 20 terms with the highest CPPs are listed in Table 3. Notable terms hinting on important issues discussed in the analyzed literature set were public perception, public concern, health authority, peer (related to peer-to-peer support), and policy maker (Table 3).

**Table 3:** Top 20 terms with the highest citations per paper.

Term^a^	Publication count (N=529), n (%)	Citations per paper
Real time	5 (0.9)	160.2
Public perception	7 (1.3)	86.4
Credible source	7 (1.3)	84.9
Public concern	11 (2.1)	75.5
Health authority	14 (2.6)	56.5
Story	24 (4.5)	54.5
Peer	11 (2.1)	50.4
Adoption	16 (3.0)	49.3
Relevant video	7 (1.3)	48.1
Term	34 (6.4)	43.3
Sentiment	19 (3.6)	41.2
Illness	6 (1.1)	41.0
Zika virus	6 (1.1)	40.3
Emergency	21 (4.0)	38.7
Policy maker	9 (1.7)	38.6
Viewer	7 (1.3)	37.1
Misperception	8 (1.5)	36.5
Information source	12 (2.3)	36.0
Feeling	6 (1.1)	35.0
Potential risk	7 (1.3)	35.0

^a^Only terms that appeared in at least 1% of papers were considered.

**Figure 3:**
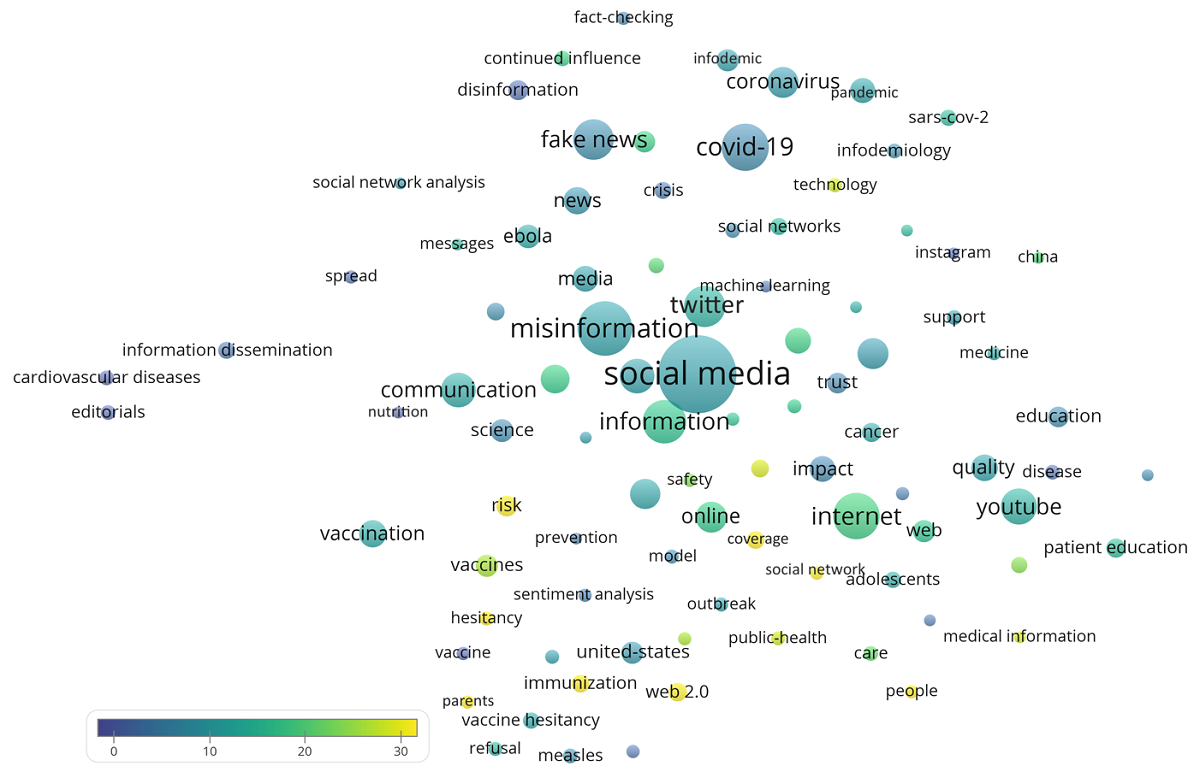
Keyword map of the 529 papers. Circle size is related to the number of papers including the word/phrase as a keyword. Circle color is related to the citations per paper. The proximity between circles is related to how frequently the terms are co-mentioned in the same papers.

A keyword map is shown in Figure 3. The keyword map showed that several diseases were recurring themes of investigation, such as measles (n=9, CPP=7.7), Ebola (n=22, CPP=11.4), COVID-19 (n=87, CPP=6.4), and cardiovascular diseases (n=9, CPP=1.7). Table 4 presents the top 20 keywords with the highest CPPs, and reveals that risk and safety were among the concepts with the highest CPPs.

**Table 4:** Top 20 keywords with the highest citations per paper.

Keyword^a^	Publication count (N=529), n (%)	Citations per paper
Risk	17 (3.2)	51.6
Social network	7 (1.3)	50.7
Parents	7 (1.3)	41.1
Hesitancy	8 (1.5)	39.5
Coverage	13 (2.5)	37.9
Immunization	13 (2.5)	34.5
People	7 (1.3)	31.0
Web 2.0	14 (2.6)	30.4
Knowledge	13 (2.5)	27.5
Medical information	6 (1.1)	27.5
Technology	8 (1.5)	27.3
Public-health	8 (1.5)	27.1
Attitudes	7 (1.3)	24.9
Vaccines	19 (3.6)	24.7
Videos	11 (2.1)	23.1
Safety	7 (1.3)	22.7
Care	9 (1.7)	20.9
Risk communication	10 (1.9)	20.0
China	6 (1.1)	19.5
Internet	86 (16.3)	18.6

^a^Only keywords that appeared in at least 1% of papers were considered.

**Figure 4:**
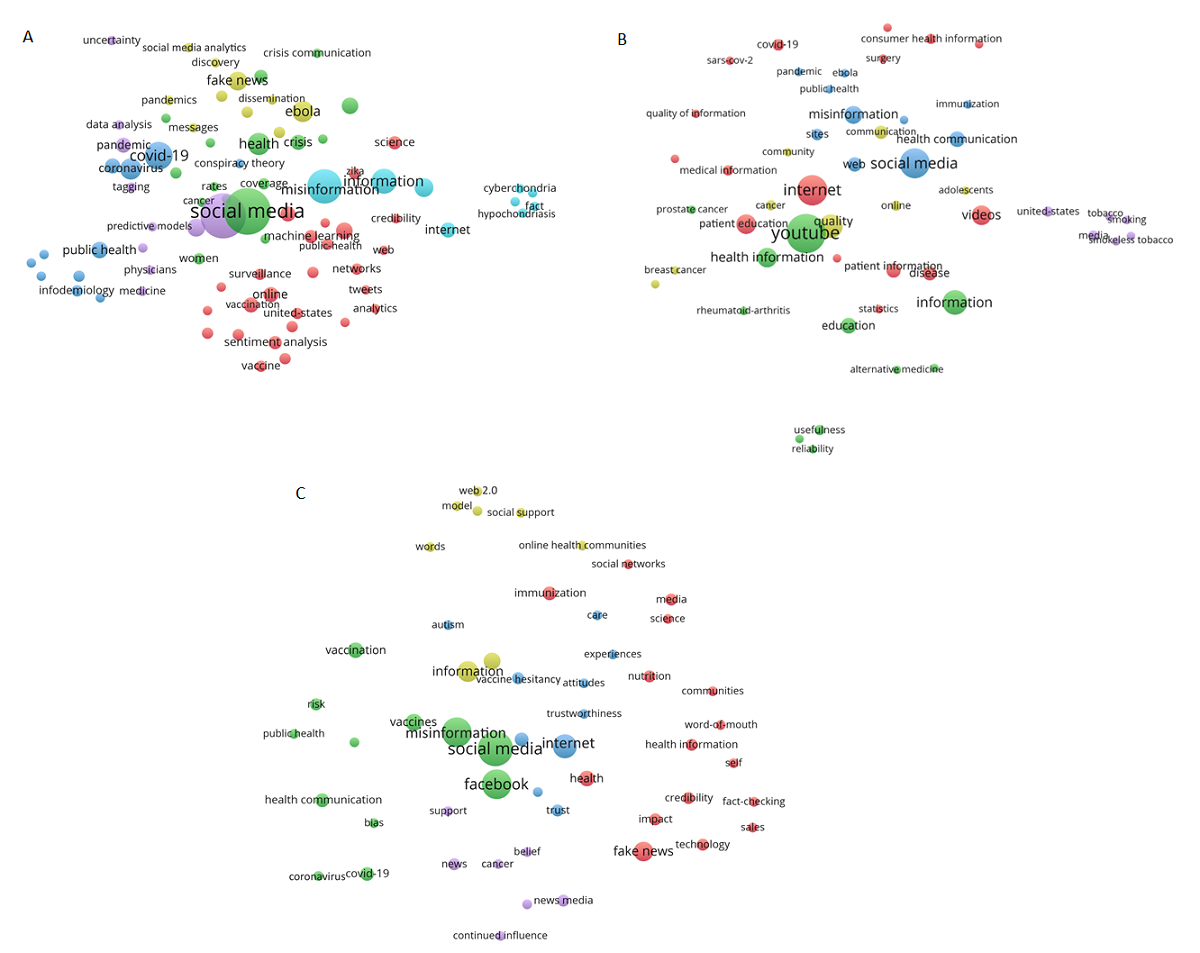
Keyword maps of the papers investigating (A) Twitter, (B) YouTube, and (C) Facebook. Circle size is related to the number of papers mentioning the respective word/phrase as a keyword. Circle color is related to the clustering of the words by the default setting of VOSviewer. The proximity between circles is related to how frequently the terms are co-mentioned in the same papers.

Keyword maps generated for the publication set that investigated Twitter, YouTube, and Facebook are shown in Figure 4A-C. The keyword maps reveal that Twitter research focused on anxiety related to online searching for disease and medical information (cyberchondria and hypochondriasis, cyan), vaccination and Zika virus (red), COVID-19 (blue), Ebola (yellow), cancer (green), and data analysis involving predictive modeling (purple). YouTube research focused on smoking and tobacco (purple), alternative medicine for various diseases, such as rheumatoid arthritis and prostate cancer (green), breast cancer (yellow), COVID-19 (red), and Ebola (blue). Finally, Facebook research focused on online health communities (yellow), vaccine hesitancy related to autism (blue), credibility of health information related to immunization and nutrition (red), cancer (purple), and COVID-19 (green).

The top 10 most cited papers are listed in Table 5. Peer-to-peer support and spread of misinformation were mentioned, and all Twitter, YouTube, and Facebook data were investigated. The themes of these top 10 papers were consistent with the list of highly cited terms listed in Table 3, covering topics such as peer-to-peer support, online videos, and public perception.

The exploratory n-gram analysis resulted in several meaningful 5-gram metatexts with at least four appearances as follows: 6 appearances, “as a source of information” and “the spread of fake news;” 5 appearances, “rumors stigma and conspiracy theories,” “the quality of health information,” and “content reliability and quality of;” 4 appearances, “health anxiety and health literacy,” “the relationship between message factors,” “intentions to trust and share,” “#PedsICU and coronavirus disease 2019,” “in low- and middle-income countries,” “actions for a framework for,” “interacted with perceived message importance,” and “verify and share the message.”

**Table 5:** Top 10 most cited papers on medical and health-related misinformation on social media.

Authors, year	Citations, n
Chew et al, 2010 [1]	613
Yaqub et al, 2014 [31]	256
Naslund et al, 2016 [32]	212
Madathil et al, 2015 [33]	195
Kamel Boulos et al, 2011 [34]	186
Betsch et al, 2012 [35]	168
Syed-Abdul et al, 2013 [36]	147
Depoux et al, 2020 [37]	136
Singh et al, 2012 [38]	123
Bode et al, 2015 [39]	121

## Discussion

### General Discussion

Using bibliometric analysis, this study identified and quantitatively analyzed 529 papers on medical and health-related misinformation on social media, revealing the most popularly investigated social media platforms, prevailing research themes, most utilized journals, and most productive countries, institutions, and authors.

### Findings Concerning the Western World and Its Prevalent Social Media Platforms

This bibliometric analysis on 529 scientific articles concerning medical and health-related misinformation on social media revealed that the most heavily investigated platforms were Twitter, YouTube, and Facebook. This could be related to the finding that most of the top 10 productive countries were from North America and Europe where these social media platforms were dominant. The results also confirmed the hypothesis that the United States had the largest contribution in social media research. The total publication and citation counts increased very rapidly especially during the last 2 years, consistent with the trends identified by previous systematic reviews on this topic [7,21]. On the other hand, this study found that original articles accounted for 74.3% of the analyzed literature set. This implied that one-fourth of the literature was not covered by the abovementioned systematic reviews, which might partly explain some differences in the results. For instance, this study found that Twitter was the most recurring social medium in the literature instead of YouTube, as reported by Wang et al [7]. The strength of the review by Suarez-Lledo and Alvarez-Galvez [21] was that it analyzed the prevalence of health misinformation posts (0%-98%) reported in the original articles. Meanwhile, Wang et al [7] categorized them into predefined theoretical frameworks with the most prevalent ones being public health, health policy, and epidemiology (n=14); health informatics (n=8); communications studies (n=5); vaccines (n=4); cyberpsychology (n=3); and system sciences (n=3). Here, it was found that publications in immunology were on average more frequently cited than communication and computer science, whereas health care sciences and medical informatics papers were in-between. This implies that the more published disciplines do not necessarily warrant more citations. This finding has 2 implications. First, quantity may not necessarily mean quality. Second, field differences in citation rates found in general [40] remained present even when the literature set was confined to a single focus on misinformation. Similar to the current findings, Wang et al [7] also found that the most popular topics were vaccination, Ebola, and Zika virus, with other less popular focus topics being nutrition, cancer, and smoking. In contrast, Wang et al [7] identified fluoridation of water as one of the recurring topics, whereas in this study, COVID-19 emerged as a strong research focus. Moreover, the performed keyword analysis in this work revealed that the research on different social media platforms, such as Twitter, YouTube, and Facebook, focused on different topics. While, at present, we do not have an explanation for this interesting observation, we believe that the reasoning for different topic studies on different social media could be a relevant direction for future research. Such studies may elucidate whether this is due to different prevalences of specific content across the platforms or due to preferential academic interest from research teams with particular interest in specific social media platforms or topics.

### Findings Concerning China and Its Prevalent Social Media Platforms

Though some social media platforms were not available in China, China still made it into the top 10 list of the most productive countries (Table 1). With a large population of internet users in China, it could be reasonably expected that Weibo and WeChat, which are popular social media platforms in China, would become more investigated in future studies. One potential barrier to non-Chinese researchers would be content translation, as the majority of their content is written in Chinese. In addition, the fast-growing short video platform TikTok (and its Chinese version Douyin) might also exert significant influence on the health information seeking behavior of internet users in the future. However, TikTok videos might be hard to archive, and video analysis tools might not be as well developed as text mining tools, which might hinder analysis by public health researchers. The same applies to the visual contents posted on Instagram. Current findings seem to suggest that sufficient research on misinformation disseminating through these platforms is missing from the current literature and should be addressed in future research. Readers should be aware that the publication bias toward Europe and North America, especially the United States, indicates that the current body of knowledge might not reflect the wider spectrum of misinformation on global health issues, especially in other parts of the world with large online communities, such as Asia and South America.

The most productive author was found to be Emily K Vraga who is based in the United States. Her studies focused on how to correct health misinformation (in other words, overturn subjects’ misperceptions) dispersed on social media, particularly Facebook and Twitter [39,41-43]. Though China was among the top 10 most productive countries, we found that only 2 of the top 50 most productive authors were based in China. They were King-Wa Fu (n=4) and Chung-Hong Chan (n=3) from the University of Hong Kong, and they focused solely on the Ebola virus [44-47]. This implied that, to grasp the research foci from China, readers need to refer to diverse works from multiple authors instead of that from a few prominent authors. With the continued growth of netizens in China, we anticipate that more productive authors might be based in China in the future.

### Elaboration on the Recurring Themes of the Literature

A very important role of social media is to provide peer-to-peer support, as investigated by some publications identified in this study (see Tables 3 and 5), for example, by forming online health communities and support groups, and ensuring stakeholder access to the latest and most relevant scientific information and health interventions [32]. For instance, users could post supportive comments and advice to YouTube videos uploaded by individuals with mental health issues [48]. On the other hand, misinformation spread via social media (especially related to Twitter, see Figure 4A) might lead to cyberchondria (cyberspace-related hypochondria; the unfounded concern escalation about common symptomology based on information found on the internet), with a study revealing that unverified information might be more easily shared by internet users who trust online information and perceived an information overload, and that women were more likely to experience cyberchondria [49]. Cyberchondria could be an important health concern, as a meta-analysis established the correlation between health anxiety and both online information seeking and cyberchondria [50], and another work revealed that it had 5 facets, including reassurance seeking and mistrust of medical professionals [51]. Being flooded by online misinformation would not alleviate the situation but may worsen it. A recent study found that government and professional videos containing solely factual information only accounted for 11% of all YouTube videos on COVID-19 and 10% of views, whereas 28% of the analyzed videos contained misinformation and had up to 62 million views [52]. In this context, the adequacy of funding and resources allocated by governmental bodies to online health literacy campaigns needs to be questioned.

Meanwhile, from the perspective of policy makers, the large amount of information from social media can be monitored and used for the achievement of efficient outcomes. As seen from the results, “health policy services” was among the most recurring journal categories for the analyzed literature set (Table 1) and “policy maker” was one of the recurring terms with the highest CPP (Table 3). For instance, by analyzing tweets related to the COVID-19 pandemic, researchers could identify the top 4 concerns of Twitter users, namely the origin, sources, impact on various levels, and ways to reduce the infection risk [53]. While using similar approaches, keeping these concerns anonymized at an individual level and ensuring that social or ethnic groups expressing specific concerns do not become targets of discrimination are crucial. Authorities could therefore focus on these concerns as they derive measures and disseminate information to the public to contain the pandemic and reduce fears within the community. In this regard, authorities could collaborate with scientific associations and provide incentives to civil society to address ignorance or misinformation on the detected concerns. Future research could compare relevant social media content following interventions, to define the optimal strategies of tackling misinformation on social media.

As mentioned in the Introduction, the fraudulent study linking the MMR vaccine to autism still has a lingering influence on social media, as it is still posted on the Facebook platform by antivaccine advocates, despite its retraction due to fraudulency [54]. Moreover, it was found that the content posted by Facebook users regarding vaccination has been increasingly polarized [20]. One study suggested that the use of angry language could promote the viral spread of the messages, including the misinformation of vaccines causing autism [55], though it was not investigating contents exclusive to vaccines and only binarized words into positive and negative emotions. Summarizing the results from n-gram analysis, netizens might need to be aware of fake news, rumors, stigma, and conspiracy theories circulating on the internet. Content reliability and quality should be assessed, and information should be verified before sharing. One way to cohort authoritative or accurate health care information shared by experts on social media (eg, Twitter) is by the use of a hashtag, so that others can search easily. One example was #PedsICU that promoted pediatric critical care content, as found by n-gram analysis. By sensible collaboration, there may be a chance to mitigate misinformation.

### Limitations

Any publications in journals not indexed by WoS were missed in the current analysis. For example, there is a relevant paper investigating misinformation of COVID-19 on TikTok, which is not indexed by WoS [56]. Besides, WoS mainly indexes papers written in English. There may be papers investigating Weibo and WeChat written in Chinese or published in Chinese journals that are not yet indexed by WoS. Preprints are also not indexed in WoS, which could be an important source of preliminary information, but the reliability of such information is debatable due to the lack of peer-review assessment. Moreover, a bibliometric study cannot assess the scientific quality of the content, such as risk of bias, effect size or statistical significance of the results, and whether the conclusions are justified by the respective data reported. The accuracy of data tagging by the literature database could also pose a limitation. For instance, KeyWords Plus are keywords tagged to a paper by an algorithm used by WoS based on the terms from the titles of the cited references [57], and are more broadly descriptive and therefore applicable to analyzing the structure of scientific fields [58]. However, it was unclear how accurate they were compared to other tags such as the National Center for Biotechnology Information’s Medical Subject Headings (“MeSH terms”). Future studies should also incorporate “conspiracy theory” and related terms into their search protocols for more comprehensive results.

### Future Research

For potential future research, artificial intelligence (AI) applications for social media content analysis would be an especially promising avenue. With increasing content and misinformation circulating on social media, it becomes practically impossible to manually determine and classify misinformation. AI or machine learning might be employed for such content analysis, which has the potential to achieve high accuracy [59]. Yet, AI could also be exploited to generate and disseminate misinformation to targeted audiences [60]. AI research in health care was most frequently published in journals in computer science and engineering, as reported by Guo et al [61], and indeed, among their identified top 10 most productive journals, only PLOS One was on the list in our study (Table 1). Along this line, with the further development of AI applications for social media content analysis, it might also be of interest to promote the dissemination of such research in mainstream public health journals, in order to reach a broader relevant audience.

### Conclusions

Based on an analysis of 529 papers on medical and health-related misinformation on social media, we found that the United States contributed to half of the papers, with 80% of the top 10 most productive institutions being based in this country. The papers were mostly published in journals belonging to the categories public environmental and occupational health, communication, health care sciences services, medical informatics, and medicine general internal. However, they were generally less cited than papers published in immunology, suggesting that more publications did not warrant more citations. Social media platform–specific papers accounted for 44% of all papers. The most popularly investigated social media platforms were Twitter, YouTube, and Facebook. They also had higher CPPs than other social media. Investigations on these platforms had different foci. Twitter-based research investigated cyberchondria and hypochondriasis, YouTube-based research investigated tobacco smoking, and Facebook-based research investigated vaccine hesitancy related to autism. COVID-19 was a common topic investigated across all platforms. An important implication of these findings is that often knowledge on specific themes related to medical misinformation relies on the predominant study of a single social media platform or limited number of platforms, and broader cross-platform studies could be a promising direction for future research. Future studies should also include social platforms aimed at non-English users to provide a wider perspective on global health misinformation.

## Appendix

Multimedia Appendix 1 PRISMA (Preferred Reporting Items for Systematic Reviews and Meta-Analysis) flow diagram of the literature search.

## Abbreviations

AI: artificial intelligence
CPPs: citations per paper
MMR: measles, mumps, and rubella
WoS: Web of Science
