# Supplementary figures and images for: Medical and Health-Related Misinformation on Social Media: Bibliometric Study of the Scientific Literature
Source: J Med Internet Res. 2022 Jan 25;24(1):e28152. doi: 10.2196/28152 (PMC8793917; doi:10.2196/28152)

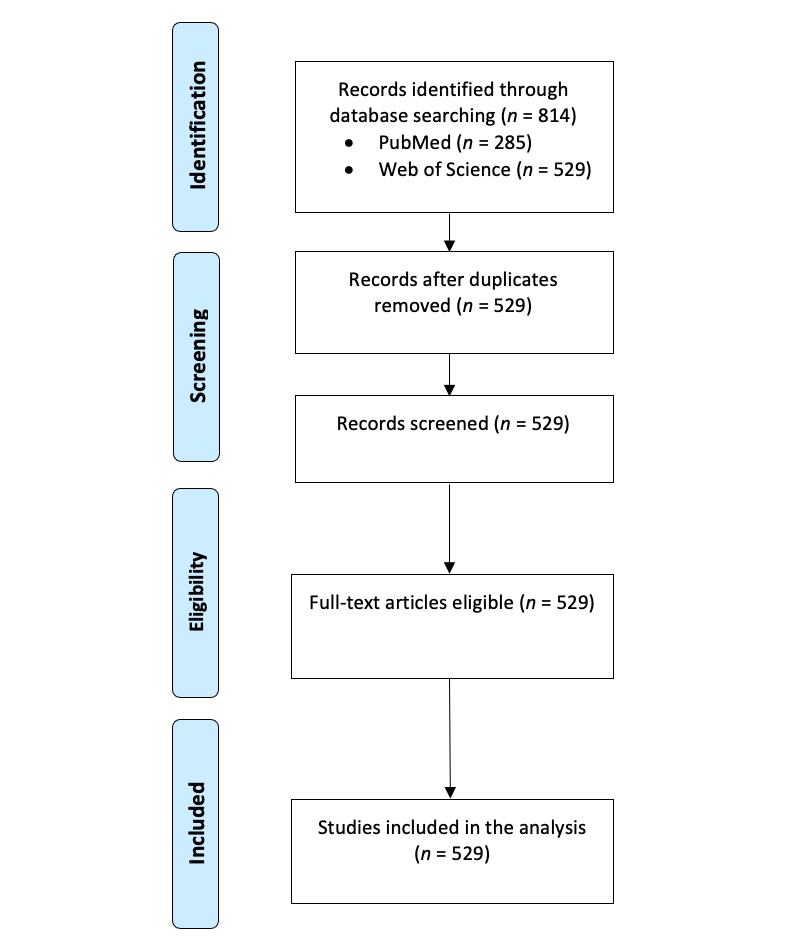

Supplement: Multimedia Appendix 1 [file jmir_v24i1e28152_app1.png]
